# Compensatory Increase of Serum Hepassocin Protects Hyperthyroidism-Induced Hepatic Dysfunction

**DOI:** 10.3390/biomedicines11071936

**Published:** 2023-07-07

**Authors:** Chih-Chen Wang, Ching-Han Lin, Hsuan-Wen Chou, Chung-Teng Wang, Yu-Cheng Liang, Hung-Tsung Wu, Horng-Yih Ou

**Affiliations:** 1 Department of Internal Medicine, National Cheng Kung University Hospital, College of Medicine, National Cheng Kung University, Tainan 70101, Taiwan; ncku054733@gmail.com (C.-C.W.); cyclops0113@yahoo.com.tw (C.-H.L.); coolpikachu2007@gmail.com (H.-W.C.); ycliang.official@gmail.com (Y.-C.L.); 2 Department of Internal Medicine, School of Medicine, College of Medicine, National Cheng Kung University, Tainan 70403, Taiwan; knight790105@hotmail.com

**Keywords:** hyperthyroidism, hepassocin, liver function, phosphoenolpyruvate carboxykinase, triiodothyronine

## Abstract

Hepatic dysfunction is commonly observed in subjects with hyperthyroidism. Hepassocin is a hepatokine playing an important role in metabolic diseases and exhibiting a hepatic protective effect. Nevertheless, the relationship between hepassocin and hyperthyroidism was still unknown. In the present study, a total of 36 subjects with Graves’ disease were enrolled, and we found that the alanine aminotransferase (ALT) levels were significantly decreased in parallel with the decrement in serum hepassocin concentrations at 6 months after standard treatment for hyperthyroidism. In addition, HepG2 cell line was used to investigate the role of hepassocin in hyperthyroidism-induced hepatic dysfunction. Treatment of hepassocin recombinant protein in HepG2 cells dose-dependently decreased triiodothyronine (T3)-induced ALT and aspartate aminotransferase (AST) elevation. Moreover, hepassocin significantly increased the expression of phosphoenolpyruvate carboxykinase (PEPCK) in a dose-dependent manner. Deletion of hepassocin in HepG2 cells reversed the effects of T3 on PEPCK expressions. Furthermore, we found that T3 increased the expression of hepassocin through a hepatocyte nuclear factor 1α-dependent pathway. Taken together, these results indicated a compensatory increase in serum hepassocin might have a protective role in hyperthyroidism-induced hepatic dysfunction.

## 1. Introduction

Thyrotoxicosis is a clinical syndrome caused by excessive thyroid hormones in circulation, while hyperthyroidism is a form of thyrotoxicosis caused by excessive endogenous production of thyroid hormones [1]. Hepatic dysfunction commonly occurs in untreated hyperthyroidism, and the prevalence of hepatic dysfunction (with at least one abnormal liver blood test) in hyperthyroidism approximately reaches 55% [2]. Despite previous studies demonstrating that improved thyroid function after treatment is typically associated with the normalization of liver function [3], the specific mechanisms underlying this phenomenon, other than the resolution of thyrotoxicosis, remain unclear due to a lack of relevant research.

It was known that hyperthyroidism promotes hyperglycemia by affecting the secretion, action, and clearance of insulin and other aspects of carbohydrate metabolism [4,5,6,7,8]. Therefore, patients with hyperthyroidism have an increased risk of hyperglycemia [9], and the prevalence of impaired glucose tolerance in the hyperthyroid state was 31.5%, which was significantly higher than that of 19.2% in healthy volunteers [8]. It is also worth noting that liver is characterized by free glucose uptake since insulin-independent glucose transporter 2 (GLUT2) enables high-capacity, facilitated diffusion of glucose into the hepatocyte [10], and hyperglycemia per se causes hepatic injury via disrupting several cellular processes, such as impaired proteosome activity and the decreased expression of molecular chaperones in the liver, promoting hepatic oxidative and nitrosative stress and disturbing cell redox potential [11,12]. Although the possible causes of liver dysfunction in hyperthyroidism have been suggested to include prolonged exposure to excessive thyroid hormones, hepatocyte anoxia, liver cell degeneration from accelerated liver glycogen and protein decomposition, autoimmune-related liver injury and congestive hepatopathy [13,14,15,16], the exact mechanisms of hyperthyroidism-related impaired liver function still remain obscure.

Hepassocin, also called fibrinogen-like protein 1 (FGL1) or hepatocyte-derived fibrinogen-related protein 1 (HFREP1), is a hepatokine within the human genome mapped to chromosome *8p22–21.3* [17]. It was first isolated in a rat model using differential cDNA expression cloning of a cDNA, and mRNA expression of this factor is upregulated in the rat liver after partial hepatectomy, suggesting its potential role as a potent mitogenic growth factor in the process of liver regeneration [18]. Hepassocin acts as a mitogen for hepatocytes and exerts a hepatic protection action against chemical-induced liver injury [19,20]. It inhibits the upregulation of proapoptotic factors (Bax, cleavage caspase 9) induced by toxins and increases the expression level of apoptotic factors (B-cell lymphoma-2 (BCL-2), B-cell lymphoma-extra large (Bcl-xL)) [20]. Previous studies also found that hepassocin not only promotes hepatocyte cell proliferation via an extracellular signal-regulated kinase 1/2 (ERK 1/2)-dependent autocrine mechanism [21] but also plays an important role in the development of obesity [22] and nonalcoholic fatty liver disease (NAFLD) [23]. Additionally, hepassocin disrupts insulin effects on gluconeogenesis in HepG2 cells via promoting the expression of phosphoenolpyruvate carboxykinase (PEPCK) and therefore increases hepatic glucose production [17,24]. Given that hyperglycemia might cause structural and functional changes in the liver [11,12], hepassocin has been shown to exert a hepatic protection effect in hyperglycemic crisis by increasing expressions of antioxidative stress proteins [25]. On the other hand, it has been previously observed that hepatocyte nuclear factor 1α (HNF 1α) plays a critical role in the regulation of hepatic hepassocin expressions [17]. However, it is still unclear whether hepassocin has a similar role of hepatic protection against hyperglycemia caused by hyperthyroidism.

Therefore, the aim of this study was to clarify the role of hepassocin in hyperthyroidism-induced hepatic dysfunction. To achieve this, we conducted a human study to observe the alterations in liver function and hepassocin levels during the treatment of Graves’ disease. Furthermore, we utilized a cell model to investigate the underlying role of hepassocin in thyrotoxicosis.

## 2. Materials and Methods

### 2.1. Human Subjects

The study protocol was approved by the Human Experiment and Ethics Committee of National Cheng Kung University Medical Center (B-ER-110-094), and all eligible subjects gave written, informed consent prior to participation. The present study was designed as a prospective cohort study conducted between August 2014 and June 2017 at the endocrinology outpatient clinic of National Cheng Kung University Hospital. Patients aged from 18 to 80 years old with newly diagnosed Graves’ disease were enrolled. The exclusion criteria included (1) women in pregnancy; (2) diabetes mellitus; (3) active liver disease; (4) renal dysfunction with serum creatinine >1.5 mg/dL; (5) congestive heart failure; (6) any acute or chronic inflammatory disease as determined by a leukocyte count >10,000 cells/mm^3^ or clinical signs of infections; (7) current usage of drugs that affect glycemia status (such as steroids); (8) malignancy; and (9) any major diseases that contraindicated this study.

Graves’ disease was defined by the presence of biochemical hyperthyroidism (elevated free tetraiodothyronine (FT4) levels (>1.79 ng/dL) and suppressed thyroid-stimulating hormone (TSH) levels (<0.05 μU/mL), together with elevated anti-thyroid stimulating hormone receptor antibodies (TRAb) or two of the following: diffuse goiter, significant (1:100 or greater) titer of thyroid peroxidase antibodies, or presence of dysthyroid eye disease.

After an overnight 12 h fast, all subjects received a blood test, including serum thyroid function tests (total triiodothyronine (T3), FT4, and TSH), serum TRAb, plasma complete blood count (CBC), fasting plasma glucose, and serum routine biochemistry parameters, including alanine aminotransferase (ALT), aspartate aminotransferase (AST), creatinine, lipid profiles (total cholesterol, triglyceride, high-density lipoprotein cholesterol (HDL-Cholesterol), and low-density lipoprotein cholesterol (LDL-Cholesterol)). All of the blood tests were determined at the central laboratory of National Cheng Kung University Hospital. All eligible patients were treated with thionamide for thyrotoxicosis. The doses of thionamide were adjusted according to FT4 levels measured before each outpatient visit to achieve normalization of serum TSH levels. Serum hepassocin concentrations were determined using commercialized enzyme-linked immunosorbent assay kits (Invitrogen, Carlsbad, CA, USA).

### 2.2. Cell Culture

HepG2 cell line was purchased from the Bioresource Collection and Research Center (Food Industry Research and Development Institute, Hsinchu, Taiwan). Cells were maintained (5% CO_2_, 37 °C) in Dulbecco’s modified eagle medium (HyClone, South Logan, UT, USA) supplemented with 10% heat-inactivated fetal bovine serum and antibiotics (100 IU/mL penicillin and 100 mg/mL streptomycin). After an overnight starvation from fetal bovine serum, the cells were treated with 3,3′,5-triiodo-L-thyronine sodium salt (Sigma-Aldrich, St. Louis, MO, USA) as T3 treatment with variable concentration and durations.

### 2.3. Lentiviral Vectors

Lentiviral vectors containing short hairpin-RNA targeted to *Homo sapiens* hepassocin or *hepatocyte nuclear factor (HNF1A) gene* and luciferase (non-targeting) were used for producing lentiviral vectors, according to our previous studies [26]. Briefly, the lentiviral particles were transfected in HEK-293T cells. After 24 h, the supernatant was collected and filtered with a 0.45 μm PTFE syringe filter. Lentiviral particles were centrifuged at 4 °C with speed of 20,000 rpm for 2.5 h. After evaluation of the titer of lentiviral solution, HepG2 was transfected with 20 multiplicity of infection (MOI) of lentiviral solution. Finally, the cells were selected by 5 μg/mL puromycin (Sigma-Aldrich), and the deletion of hepassocin or HNF1α was confirmed by Western blot analyses.

### 2.4. Western Blot Analyses

The samples were lysed with a buffer containing 150 mM NaCl, 1% Triton X-100 (polyethylene glycol octyl phenyl ether), 100 mM tris (hydroxymethyl)-aminomethane (Tris)-HCl (pH 8.0)), 1 mM ethylenediaminetetraacetic acid (EDTA), 1 mM ethylene glycol tetraacetic acid (EGTA), 0.2 mM sodium orthovanadate, 0.2 mM phenylmethylsulfonyl fluoride (PMSF), and 0.5% Nonidet P-40. The cell lysates were collected, and the protein levels were quantified using a bicinchoninic acid protein assay kit (Thermo Scientific, Rockford, IL, USA). The cell lysates were boiled at 95 °C in sample buffer for 7 min to perform denaturation and then electrophoresized in a running buffer containing 25 mM Tris base, 192 mM glycine, and 1% sodium dodecyl sulfate (SDS) (pH 8.3). Proteins from the lysates were then resolved by a buffer containing 25 mM tris and 192 mM glycine (pH 8.3) and transferred under 70 voltages for 90 min onto polyvinylidene fluoride membranes (Millipore, Billerica, MA, USA). After blocking by Tris-buffered saline with Tween 20 detergent (TBST) containing 10% skim milk at room temperature for 1 h, the blots were probed with primary antibodies, including hepassocin (Proteintech Group, Rosemont, IL, USA), PEPCK (Santa Cruz, CA, USA), actin (Millipore, Billerica, MA, USA), HNF-1α (Novus Biologicals, Littleton, CO, USA), Histone deacetylase 1 (HDAC1) (Abcam, Cambridge, UK), and glyceraldehyde 3-phosphate dehydrogenase (GAPDH) (Abcam) at 4 °C overnight. After the membranes had been washed with TBST, the blots were incubated with a 1:5000 dilution of horseradish peroxidase-conjugated secondary antibodies at room temperature for 1 h, and then washed with TBST. The protein bands were detected using Immobilon (Millipore), and the optical density of the protein levels was determined using VisionWorks LS software 7.0 (UVP, LLC, Upland, CA, USA).

### 2.5. Statistical Analysis

SPSS software (version 17.0; IBM Corp., Armonk, NY, USA) was used for statistical analysis. All normally distributed continuous variables were expressed as means ± standard deviation (SD) for human study. A paired *t*-test was employed to compare the difference in ALT and hepassocin before and after treatment. The relationships between hepassocin, T3, FT4, and fasting plasma glucose were examined using Spearman correlation analysis. In cell study, the results were expressed as means ± standard error of the mean (SEM) and analyzed by analysis of variance (ANOVA). A *p* value less than 0.05 was considered statistically significant.

## 3. Results

### 3.1. Serum Hepassocin Concentrations Were Significantly Decreased in Subjects with Graves’ Disease Received Standard Treatment

**Figure 1:**
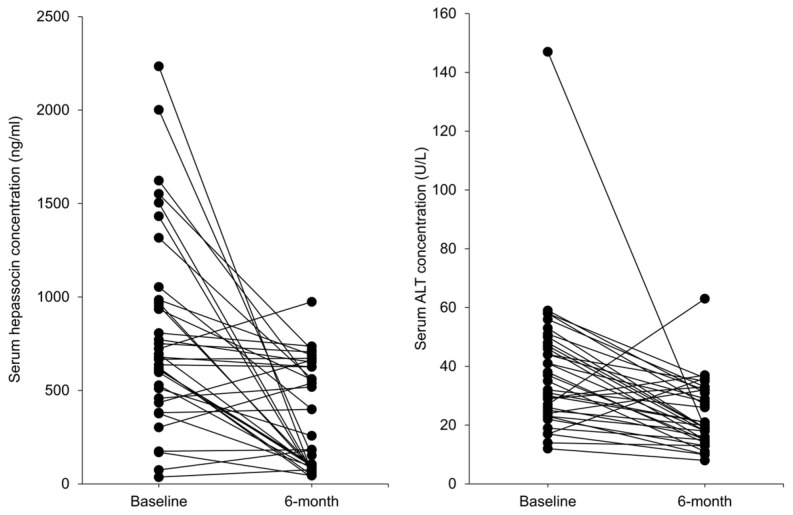
Decrement in serum hepassocin concentrations accompanied with improved hepatic function in subjects with hyperthyroidism. A total of thirty-six subjects with hyperthyroidism were enrolled and the serum samples were collected for the determination of serum hepassocin and alanine aminotransferase (ALT) concentrations at diagnosis (Baseline), and six months after the standard treatment of hyperthyroidism.

In order to investigate the changes of hepassocin in subjects with Graves’ disease, a total of 36 study subjects, including 9 males and 27 females, were enrolled in the present study. Table 1 shows the baseline characteristics of the enrolled subjects at diagnosis of Graves’ disease before thionamide treatment. At baseline, hepatic function indices of the study subjects, including ALT and AST, fell at the upper limit of the normal range (38.4 ± 23.0 U/L and 28.9 ± 15.9 U/L, respectively). At 6 months after thionamide treatment, the levels of ALT were decreased significantly (38.4 ± 23.0 to 23.1 ± 10.9 U/L, *p* = 0.001), in parallel with a decrease in serum hepassocin concentrations (799.99 ± 513.71 to 347.15 ± 282.31 ng/mL, *p* < 0.001) (Figure 1). In addition, using correlation analysis, we found there were significantly positive correlations of hepassocin with T3 (r = 0.375, *p* = 0.001), FT4 (r = 0.438, *p* < 0.001), and fasting plasma glucose (r = 0.235, *p* = 0.047).

### 3.2. Treatment of T3 Dose-Dependently Increased Hepassocin and PEPCK Expressions in HepG2 Cells

**Figure 2:**
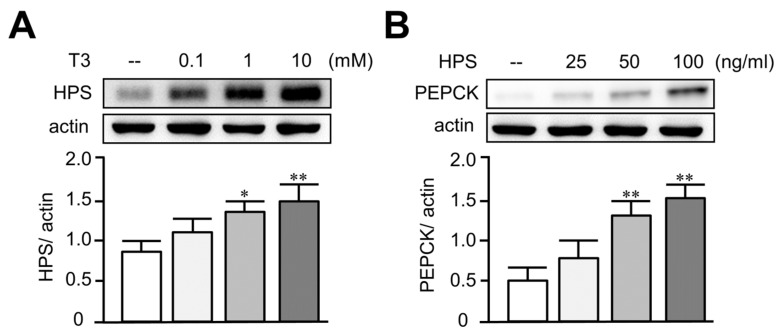
Triiodothyronine increased hepassocin expression and hepassocin-induced phosphoenolpyruvate carboxykinase expression in HepG2 cells. HepG2 cells were treated with triiodothyronine (T3) at indicated doses for 24 h, and the cell lysates were collected for the determination of hepassocin (HPS) expressions by Western blots (**A**). HepG2 cells were treated with HPS recombinant protein at indicated concentrations for 24 h, and the cell lysates were collected for the determination of phosphoenolpyruvate carboxykinase (PEPCK) expressions by Western blots (**B**). * *p* < 0.05; ** *p* < 0.01 as compared with control group.

To investigate the causal relationship of hepassocin and thyrotoxicosis, a HepG2 cell model was used. Treatment of T3 in HepG2 cells significantly increased the expression of hepassocin in a dose-dependent manner (Figure 2A). Given that a previous study indicated that hepassocin has the effect of inducing insulin resistance [26], we investigated the effect of hepassocin on gluconeogenesis. As shown in Figure 2B, we found that treatment of hepassocin recombinant protein in HepG2 cells significantly increased the expression of PEPCK in a dose-dependent manner.

### 3.3. Hepassocin Reversed T3-Induced Hepatic Enzyme Elevation in a Dose-Dependent Manner

**Figure 3:**
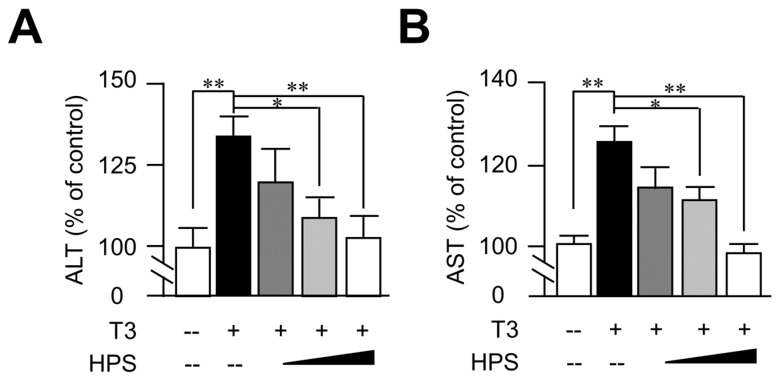
Treatment of hepassocin recombinant protein reversed triiodothyronine-induced hepatic enzyme release in HepG2 cells. HepG2 cells were pre-treated with 100 ng/ml hepassocin (HPS) recombinant protein for 1 h and then treated with triiodothyronine (T3) for another 24 h. The supernatants of each group were collected for the determination of alanine aminotransferase (ALT) (**A**) and aspartate aminotransferase (AST) (**B**) using commercialized assay kits. * *p* < 0.05; ** *p* < 0.01 as compared with control group.

In addition to the role of hepassocin in the development of insulin resistance, it was known that hepassocin has an effect against hyperglycemia-induced hepatic dysfunction. [25]. We further investigated the effects of hepassocin on liver function indices. As shown in Figure 3, pretreatment with hepassocin recombinant protein significantly reversed T3-induced AST and ALT levels in the supernatants of the HepG2 cells culture medium.

### 3.4. Knockdown of Hepassocin Diminished the Effects of T3 on PEPCK Expression

**Figure 4:**
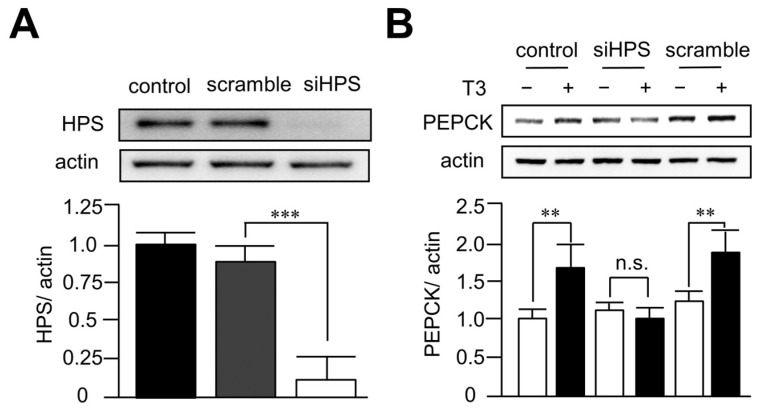
Deletion of hepassocin reversed the effects of triiodothyronine on phosphoenolpyruvate carboxykinase expression in HepG2 cells. HepG2 cells were transduced with a lentiviral vector containing short hairpin-RNA targeted to hepassocin (HPS) for 48 h to knockdown HPS (**A**). The cells were then treated with 10 mM triiodothyronine (T3) for another 24 h, and the cell lysates were harvested for the determination of phosphoenolpyruvate carboxykinase (PEPCK) by Western blots (**B**). ** *p* < 0.01 and *** *p* < 0.001 as compared with indicated groups. n.s.: not significant.

In order to investigate the role of hepassocin in hyperthyroidism-induced hyperglycemia, hepassocin was knocked down in HepG2 cells using a lentiviral vector containing short hairpin-RNA targeted to hepassocin. As shown in Figure 4A, the knockdown efficiency of hepassocin reached > 90% in HepG2 cells. In addition, deletion of hepassocin significantly diminished the effects of T3 on the expression of PEPCK (Figure 4B), implying hepassocin might be involved in T3-induced hepatic gluconeogenesis.

### 3.5. T3 Increased Hepassocin Expression through an HNF-1α-Dependent Pathway in HepG2 Cells

**Figure 5:**
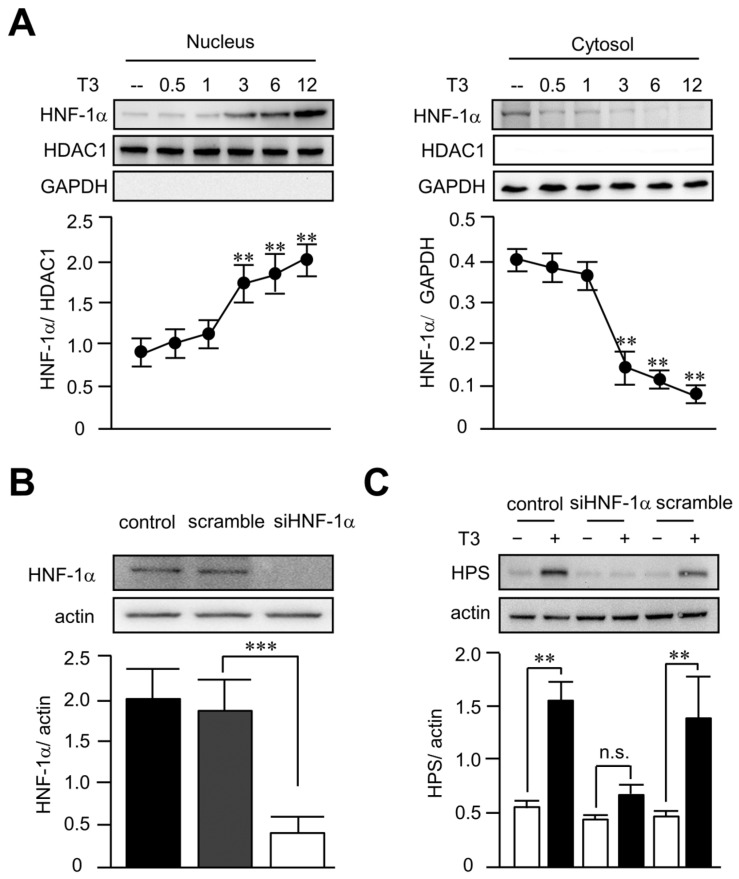
Treatment of triiodothyronine increased hepatocyte nuclear factor-1 alpha nucleus translocation and hepassocin expression in HepG2 cells. HepG2 cells were treated with triiodothyronine (T3), and the cell lysates were harvested at indicated times. The cytosolic and nuclear protein fractions were separated using commercialized kits for the determination of hepatocyte nuclear factor-1α expressions (HNF-1α) by Western blots (**A**). HepG2 cells were transduced with a lentiviral vector containing short hairpin-RNA targeted to HNF-1α for 48 h to knockdown HNF-1α (**B**). The cells were then treated with 10 mM triiodothyronine (T3) for another 24 h, and the cell lysates were harvested for the determination of hepassocin (HPS) expression by Western blots (**C**). ** *p* < 0.01 and *** *p* < 0.001 as compared with the untreated group or indicated groups. n.s.: not significant.

Given that HNF1 binding site and HNF-1α are critical to hepassocin expressions in the liver [17], we then investigated the effects of T3 on the activity of HNF-1α. As shown in Figure 5A, we found that treatment of T3 in HepG2 cells significantly increased the nucleus translocation of HNF-1α at 3 h, implying HNF-1α might be involved in T3-induced hepassocin expression. In order to confirm the role of HNF-1α in T3-induced hepassocin expression, knockdown of HNF-1α was achieved using a lentiviral vector containing short hairpin-RNA targeted to HNF-1α. As shown in Figure 5B, the knockdown efficiency of HNF-1α reached > 80% in HepG2 cells. In addition, deletion of HNF-1α significantly diminished the effects of T3 on the expression of hepassocin (Figure 5C), indicating T3 increased hepassocin expression through an HNF-1α-dependent pathway in HepG2 cells.

## 4. Discussion

Liver function abnormalities are commonly observed in patients with newly diagnosed hyperthyroidism [2,27]. Although recovery of liver function accompanies treatment of hyperthyroidism [2,3], the exact mechanisms are still unclear. To the best of our knowledge, this study represents the first attempt to investigate the hepatic protective effect of hepassocin in the context of hyperthyroidism. Our findings not only demonstrated normalization of ALT in parallel with a significant decrease in hepassocin after treatment for hyperthyroidism, but they also revealed the regulations of hepassocin expression under thyrotoxicosis using a HepG2 cell model. Thus, we clarified the role of hepassocin in thyrotoxicosis-induced hepatic dysfunction and provided valuable information to elucidate the underlying mechanisms.

According to the systematic review by Scappaticcio et al. [2], the degree of elevation of hepatic transaminase in hyperthyroidism was mostly borderline (< 2 folds of the upper limit of normal (ULN) or mild (2~5 × ULN)). In addition, the prevalence of resolution in abnormal liver blood tests was as follows: ALT, 83% (confidence intervals, CI, 72–90%); AST, 87% (CI 74–94%); alkaline phosphatase, 53% (CI 32–73%); bilirubin, 50%; and γ-glutamyltransferase, 70% (CI 47–87%), along with the restoration of euthyroidism via antithyroid drug therapy [2]. In terms of recovery time, previous studies stated that the normalization time varied from 6 weeks [28] to 12 months [29]. Similarly, our study observed that the values of liver function indices of the enrolled subjects reached the ULN but displayed a significant decrement in 6 months after treatment, which is consistent with the previous studies.

Although it was known that hepassocin has a hepatic protective effect against chemical-induced liver injury [19,20], the effects of hepassocin on thyrotoxicosis-induced impaired liver function remained unclear. It had been established that daily injections of triiodothyronine induced hepatic dysfunction related to hepatic apoptosis in rats [30]. Similarly, we found that the elevated liver transaminase induced by T3 in HepG2 cells was dose-dependently improved by hepassocin, and thus elucidated that hepassocin has an effect of improving hyperthyroidism-induced hepatic dysfunction. It has been suggested that increased oxygen consumption, a result of the enhanced metabolic rate, is considered to be a basic mechanism that leads to hepatic apoptosis and oxidative stress. This therefore induces liver abnormalities, including increases in ALT and AST levels, in thyrotoxic patients [31]. In this respect, hepassocin has been shown to increase the expressions of antioxidative stress proteins including superoxide dismutase 1 and glutathione peroxidase in an animal study of hyperglycemic mice [25]. This might also account for its possible hepatic protective effect in hyperthyroidism.

According to the previous study [32], hepassocin is an acute-phase reactant that dose- dependently responds to interleukin 6 (IL-6), which depends on the signal transducer and activator of transcription 3 (STAT3) and HNF-1 binding sites in the hepassocin promoter to induce the promoter activity of hepassocin [17]. Using the HepG2 cell model, we successfully demonstrated that T3 treatment increased hepassocin and PEPCK expressions in a dose-dependent manner. This finding offers substantial evidence to support our speculation that decrease in hepassocin levels, accompanied with improvement of thyrotoxicosis in humans and changes in serum hepassocin levels, may be a reactive and compensatory response to thyrotoxicosis. Additionally, high glucose regulates hepassocin expression in an HNF1- and STAT3-dependent manner [25], and serum hepassocin concentrations were significantly decreased after treatment of hyperglycemia in patients with hyperglycemic crisis. Since hyperglycemia resulting from hyperthyroidism is considered reversible after treatment of thyrotoxicosis [33,34], the change in plasma glucose level might also partially account for the decreased expression of hepassocin.

In the current study, T3 increased the hepassocin and PEPCK expressions in HepG2 cells, whereas deletion of hepassocin in HepG2 cells diminished T3-induced PEPCK expression, indicating hepassocin plays a role in hyperthyroidism-induced hyperglycemia. In human studies, fasting glucose concentrations are positively associated with circulating hepassocin levels [26,35]. On the other hand, overexpression of hepassocin in the liver induces insulin resistance and hyperglycemia in mice, while knockdown of hepassocin reverses insulin resistance in diabetic mice [26]. To some extent, the significant decrease in fasting plasma glucose in our cohort after treatment of hyperthyroidism (108.0 ± 19.6 to 91.2 ± 8.4 mg/dL, *p* < 0.001) might also reflect the alteration of serum hepassocin levels.

HNF-1α was known as a critical factor that regulated the expression of hepassocin in the liver [17], and high glucose induced hepassocin expression through the signal transducer and activator of transcription 3-protein phosphatase 2A-HNF1 pathway in HepG2 cells. In our study, we revealed that T3 induced the translocation of HNF-1α to increase the expression of hepassocin in HepG2 cells. Thyrotoxicosis and hyperglycemia increased hepassocin expression via the HNF-1α pathway, promoting PEPCK expression and leading to gluconeogenesis and hyperglycemia. Consequently, compensatory increase in hepassocin might play a protective role against hepatic dysfunction induced by hyperthyroidism.

There are some limitations in this study. First, the number of participants was relatively small owing to the limited availability of hepassocin data. Although we demonstrated that hepassocin exerts a protective effect on hyperthyroidism-induced hepatic dysfunction in a HepG2 cell model, future human studies with a larger sample size are still needed to investigate the effects of hepassocin on hyperthyroidism-induced hepatic dysfunction and hyperglycemia. Second, even though we revealed a parallel decrement of ALT and hepassocin in human subjects, further study on the serial changes of hepassocin to confirm its release pattern during the clinical course of hyperthyroidism will be needed. Third, there are certain specific ingredients in food that affect thyroid function tests [36,37]. Although our cohort is well-established for Graves’ disease with biochemical hyperthyroidism, along with typical signs and symptoms and elevation of TRAb, the eating habits were not recorded in the present study.

In conclusion, our work suggested that hepassocin might have a protective role in hyperthyroidism-induced hepatic dysfunction. Thyroid hormone increased hepassocin expression via HNF-1α pathway and therefore promoted gluconeogenesis. Moreover, compensatory increase in hepassocin in patients of hyperthyroidism might offset the deleterious effects of thyrotoxicosis-induced hepatotoxicity.

## Figures and Tables

**Table 1:** Baseline characteristics of study subjects at diagnosis of Graves’ disease.

Characteristic	
*n*	36
Age (y)	37.9 ± 11.5
Sex (male/female)	9/27 (25/75)
Body weight (kg)	55.98 (9.46)
BMI (kg/m^2^)	21.72 (2.79)
Systolic blood pressure (mmHg)	124.2 (13.3)
Diastolic blood pressure (mmHg)	71.8 (11.2)
Fasting plasma glucose (mg/dL)	108.0 (19.6)
Creatinine (mg/dL)	0.42 (0.18)
eGFR (mL/min/1.73 m^2^)	89.2 (5.0)
ALT (U/L)	38.4 (23.0)
AST (U/L)	28.9 (15.9)
TSH * (μU/mL)	<0.03 (<0.03 ~ <0.03)
Total T3 (ng/dl)	371.24 (158.57)
Free T4 (ng/dL, mean)	3.45 (0.91)
TRAb (U/L)	12.11 (12.34)
Hepassocin (ng/mL)	799.99 (513.71)
Total cholesterol (mg/dL)	173.1 (33.0)
Triglyceride (mg/dL)	93.0 (32.5)
HDL-c (mg/dL)	63.9 (13.5)
LDL-c (mg/dL)	104.8 (30.2)

Data are expressed as mean (SD) or * median (interquartile range) for continuous variables and *n* (%) for categorical variables. Abbreviations: body mass index, BMI; estimated glomerular filtration rate, eGFR; alanine aminotransferase, ALT; aspartate aminotransferase, AST; thyroid-stimulating hormone, TSH; total triiodothyronine, Total T3; free thyroxine, Free T4; anti-thyroid stimulating hormone receptor antibody, TRAb; high-density lipoprotein cholesterol, HDL-c; low-density lipoprotein cholesterol, LDL-c.

## Data Availability

The data that support the findings of this study are available from the corresponding author upon reasonable request.
